# Differential Proinflammatory and Oxidative Stress Response and Vulnerability to Metabolic Syndrome in Habitual High-Fat Young Male Consumers Putatively Predisposed by Their Genetic Background

**DOI:** 10.3390/ijms140917238

**Published:** 2013-08-22

**Authors:** Pedro González-Muniesa, María Pilar Marrades, José Alfredo Martínez, María Jesús Moreno-Aliaga

**Affiliations:** 1 Department of Nutrition, Food Sciences and Physiology, University of Navarra, 31008 Pamplona, Spain; E-Mails: pgonmun@unav.es (P.G.-M.); pmarrades@unav.es (M.P.M.); jalfmtz@unav.es (J.A.M.); 2 CIBERobn Physiopathology of Obesity and Nutrition, Centre of Biomedical Research Network, 29029 Madrid, Spain

**Keywords:** metabolic syndrome, microarray, inflammation, oxidative stress, subcutaneous adipose tissue

## Abstract

The current nutritional habits and lifestyles of modern societies favor energy overloads and a diminished physical activity, which may produce serious clinical disturbances and excessive weight gain. In order to investigate the mechanisms by which the environmental factors interact with molecular mechanisms in obesity, a pathway analysis was performed to identify genes differentially expressed in subcutaneous abdominal adipose tissue (SCAAT) from obese compared to lean male (21–35 year-old) subjects living in similar obesogenic conditions: habitual high fat dietary intake and moderate physical activity. Genes involved in inflammation (*ALCAM*, *CTSB*, *C1S*, *YKL-40*, *MIF*, *SAA2*), extracellular matrix remodeling (*MMP9*, *PALLD*), angiogenesis (*EGFL6*, *leptin*) and oxidative stress (*AKR1C3*, *UCHL1*, *HSPB7* and *NQO1*) were upregulated; whereas apoptosis, signal transcription (*CITED 2* and *NR3C1*), cell control and cell cycle-related genes were downregulated. Interestingly, the expression of some of these genes (*C1S*, *SAA2*, *ALCAM*, *CTSB*, *YKL-40* and *tenomodulin*) was found to be associated with some relevant metabolic syndrome features. The obese group showed a general upregulation in the expression of inflammatory, oxidative stress, extracellular remodeling and angiogenic genes compared to lean subjects, suggesting that a given genetic background in an obesogenic environment could underlie the resistance to gaining weight and obesity-associated manifestations.

## 1. Introduction

Inflammation is nowadays considered as a key feature associated to fat accumulation and obesity related conditions [1]. This pro-inflammatory status seems to be initially located in white adipose tissue, being currently suggested to be largely related to a dysregulation in adipokine secretion, which leads to different pathological conditions associated with obesity and metabolic syndrome features such as type 2 diabetes and cardiovascular disease [2–4]. Three reasons have been proposed by different groups to explain this inflammatory process occurring within adipose tissue: (a) local hypoxia [5,6]; (b) endoplasmic reticulum stress [7–9]; and (c) oxidative stress [10–12].

The prevalence of obesity is rising worldwide, which is likely to be a consequence of changes in modern societies, where easy and cheap availability of high-calorie yielding foods is combined with a sedentary lifestyle [13,14]. In this context, it has been suggested that the inflammatory state associated with obesity appears to be predominantly triggered by excessive nutrient intake [15] and/or unhealthy dietary patterns [16]. Further to this, a human study with almost 3000 people has shown that subjects with higher concentrations of inflammatory markers in their blood are more prone to gaining weight [17]. However, in this condition of chronic disturbance of metabolic homeostasis, some subjects seem to be more resistant to gaining weight and to showing metabolic syndrome manifestations [18,19]. Thus, there are consistent evidences from different human studies about the importance of the individual genetic background in the fat deposition and in the success of weight loss programs [14,20,21].

Indeed, a study carried out in mice revealed that the inflammatory state associated with obesity appears to be partly triggered by high fat diet and excessive weight [22]. Furthermore, the composition and quantity of the fat content of a meal seems to be directly related to the magnitude of the postprandial inflammatory response [23]. In this context, adipose tissue is one of the organs responsible for nutrient clearance from blood [24]. Therefore, it is reasonable to suggest a main role of inflammation in the vulnerability to obesity and the metabolic syndrome [25,26].

Thus, a genetic background favoring a pro-inflammatory status, in the presence of increased food availability could underlie the predisposition to develop obesity [27]. In order to clarify differences in the functional capacity of the adipose tissue, a pathway analysis was performed to identify inflammatory and metabolic genes differentially expressed in obese *vs.* lean subjects living in similar obesogenic conditions that could underlie in the vulnerability to obesity and metabolic syndrome development. Indeed, the interest of this trial was the fact that some subjects consuming the same amount of fat/energy and showing similar physical activity patterns produced different body weight phenotypes since some of them were obese and others were lean, which could be, at least in part, related to differences in the gene expression profile in white adipose tissue.

## 2. Results and Discussion

### 2.1. Baseline Characteristics of Lean and Obese Subjects

Descriptive characteristics at baseline of lean and obese subjects with similar habitual dietary intake of fat (>40%) and moderate physical activity are reported in Table 1. As expected, waist circumference was significantly higher in the obese than in lean subjects. Insulin sensitivity revealed by Quantitative Insulin-Sensitivity Check Index (QUICKI) was significantly lower in obese compared to lean subjects. The fasting lipid profile including Total Cholesterol and Total Cholesterol/HDL (High-Density Lipoprotein)-Cholesterol ratio was significantly higher (*p* < 0.01) in the obese compared to lean persons. The systolic and diastolic blood pressure values were significantly elevated (*p* < 0.01) in the obesity condition. Despite that high fat diet is associated with the occurrence of metabolic syndrome manifestations, lean volunteers showed no features of metabolic syndrome. Five of the obese volunteers were considered as obese with metabolic syndrome (WHO), based on the presence of three or more of the following characteristics according to the National Cholesterol Education Program: waist circumference greater than 102 cm; blood pressure of at least 130/85 mmHg; serum glucose level of at least 110 mg/dL; serum triacylglycerol level of at least 150 mg/dL; and HDL-cholesterol level of less than 40 mg/dL.

One group of lean subjects that despite showing a high fat intake and moderate physical activity remained lean and resistant to weight gain and with no features of metabolic syndrome was identified, which should be attributed to different genetic make-up [18]. The failure of the adipose tissue to buffer postprandial lipids due to a metabolic inefficacy, has been suggested as a mechanism triggering inflammatory response in adipose tissue [28]. Thus, several studies have recently shown a cross-talk between metabolic and immune system and how important this link could be to the development of obesity and/or its co-morbidities [29–31]. Mitochondrial dysfunction in adipocytes due to an excessive free fatty acid release and local hypoxia, common features in the adipose tissue from obese patients, seems to induce insulin resistance and lipotoxicity [32,33]. In fact, the mitochondrion gene ontology (GO) category (cellular component) was downregulated in obese compared to lean (*p* < 0.001).

### 2.2. Over-Represented GO Biological Process Categories

#### 2.2.1. Genes Involved in Inflammation

Pathway analysis revealed that the most notable class of genes upregulated in subcutaneous abdominal adipose tissue (SCAAT) of obese compared to lean subjects concerned the immune response (12 genes *p* < 0.001, Table S1). This category included genes encoding members for the Complement system, as *C1S*, *CD163* and *CD59*; Antigen processing: HLA-DQA1, HLA-DRB4 and CTSB; Genes involved in T cell response including: *SPP1*, *DEFA1*, *SAA1*, *SAA2* and *ALCAM*. In addition, MIF a chemotactic factor for monocytes/macrophages and YKL-40, a human glycoprotein, were also upregulated. In order to validate the results of the microarray, the gene expression upregulation of *ALCAM*, *CTSB*, *C1S*, *YKL-40*, *MIF* and *SAA2* was verified by Real Time-Polymerase Chain Reaction (RT-PCR, Table 2).

Moreover, a downregulation of some similar genes involved in inflammation has been reported after weight loss [34], which clearly ameliorates the cardiovascular risk and metabolic syndrome features. Furthermore, *MIF* has been reported to be upregulated in adipocytes exposed to 1% O_2_ [35].

We reported for the first time the upregulation of *activated leukocyte-cell adhesion molecule (ALCAM*), a broadly expressed adhesion molecule of the Ig superfamily, which shows high sequence homology with one candidate HDL receptor, HB2-high-density lipoprotein-binding protein 2, one of a pair of liver HDL binding proteins [36]. Additionally, we also found that *YKL-40* gene expression appeared overexpressed. *YKL-40* has been identified as a biomarker of inflammation, as it is elevated in patients with type 2 diabetes and related to insulin resistance [37] and extracellular matrix (ECM) remodeling [38], and is also elevated in cardiovascular disease [39]. Moreover, YKL-40 was described as being secreted by adipose tissue [40]. In accordance to our data, Hempen *et al.* [41] also observed that YKL-40 is elevated in morbidly obese patients, and declines after weight loss. However, Nielsen *et al.* [42] found that YKL-40 is an obesity-independent marker of type 2 diabetes.

Interestingly, the most upregulated gene in the array, *HLA-DRB4*, has neither been previously reported to be expressed in the adipose tissue, nor related with obesity. Due to its role in the immune system [43] it can be hypothesized that its different expression might be explained by the infiltration of macrophages occurring in obese subjects, although this needs to be clarified in future experiments.

It is well known that the expanding adipose tissue during high fat feeding makes a substantial contribution to the development of obesity-linked inflammation via dysregulated production of pro-inflammatory cytokines (such as TNF-alpha and Interleukin-6) [44], chemokines and adipokines and the reduction of anti-inflammatory adipokines (like adiponectin). In this context, we found a significant upregulation of *IL-6* mRNA (1.00 ± 0.12 *vs.* 2.82 ± 0.55, *p* < 0.05) and a downregulation of *adiponectin* [45] in SCAAT of our obese volunteers. This state of chronic low-grade inflammation could be powerfully augmented through the infiltration of macrophages into white adipose tissue, which perpetuate a proinflammatory vicious cycle [46]. Taking all present data together, it can be hypothesized that adipose tissue itself is involved in the chronic activation of relatively nonspecific defence system, as other groups have proposed [29,30]. Thus, a genetic background favoring a chronic disturbance of the metabolic homeostasis could lead to an upregulation of the proinflammatory-related genes, which could underlie the development of the metabolic syndrome.

#### 2.2.2. Genes Involved in Extracellular Matrix Remodeling

Cell adhesion (*p* < 0.01) and proteolysis (*p* < 0.05) pathways both involved in extracellular matrix (ECM) remodeling showed higher mRNA levels of genes encoding for focal adhesion and ECM: *CTGF*, *LTBP2*, *ITGB5*, *SPON2*, *MMP9* and *WISP2*, whereas *TIMP4* and *PTENP1* two inhibitors were downregulated in SCAAT of obese subjects. Interestingly, the expression of genes encoding a range of proteins associated with cytoskeletal structure of cells as *Transgelin*, *TUBB2* and *PALLD* were upregulated (Table S1).

Therefore, obese volunteers showed an upregulation in some extracellular matrix remodelation-related genes, a process that is suggested to take place during obesity to accommodate adipose tissue expansion, and which seems to be very important in the development of obesity and its co-morbidities [47]. Different studies in animals and humans have shown that some ECM-related genes are upregulated, such as *osteopontin* [48]. However, other genes like the *MMP*s (*matrix metalloproteinases*), seem to be downregulated [49,50]. Furthermore, an upregulation of *PALLD*, a novel actin cytoskeleton-associated protein, essential for cell-ECM interaction through maintaining normal actin cytoskeleton architecture [51], was detected. Recently, the possible relation of this gene with myocardial infarction [52] and pancreatic cancer [53] has been discarded, but a possible link with obesity is still under research. Another group of researchers observed, by DNA microarray, an expression of this gene of more than eight fold higher in large adipocytes compared to small adipocytes [54]. More information is needed to fully understand these outcomes.

#### 2.2.3. Genes Involved in Angiogenesis

Our data show that the expression of several proangiogenic factors was also upregulated (*p* < 0.05) in obese subjects. These factors included *EGFL6*, *Leptin*, *CTGF* and *cysteine-rich protein-61* (*CYR61*) (Table S1). In addition, ALCAM [55] and CTSB [56] also involved in inflammatory processes had angiogenic activities.

In this context, accumulating evidence suggests that adipose tissue growth/expansion is dependent on angiogenesis and endothelial cell proliferation [57]. Here, we demonstrated that *EGFL6*, an angiogenesis-related gene previously shown to be expressed in human adipose tissue [58], is upregulated in the obese. In addition, *CTSB* a novel cathepsin member, as *CTSK* and *CTSP* previously related with inflammation in White Adipose Tissue (WAT) [59,60], which encodes a lysosomal protease implicated in degradation of ECM and angiogenesis [61] was upregulated in obese compared to lean subjects. Interestingly, *CTSB* has been found to be overexpressed and more active under hypoxic conditions [62], as those suggested to occur in obese patients [5]. However, our data revealed that *tenomodulin*, which is considered a putative angiogenesis inhibitor, was also found to be upregulated in obese subjects. This is in agreement with the results from Saiki *et al*. [63]. Other investigations have highlighted the importance of polymorphisms in this gene in the development of obesity and type 2 diabetes [64], cholesterol metabolism [65] and mild inflammation [66]. Furthermore, Kolehmainen *et al*. [67], suggested that *tenomodulin* could be involved in extracellular matrix remodeling, and in these samples this might be its main role instead of its anti-angiogenic properties.

#### 2.2.4. Genes involved in Oxidative Stress

The analysis of the microarray revealed that several genes encoding proteins involved in ROS (Reactive oxygen synthesis) activity were overexpressed, such as *AKR1C3*, *UCHL1*, *HSPB7* and *NQO1*.

Oxidative stress is considered one of the main reasons triggering and maintaining the inflammatory processes that occur within obesity and related co-morbidities, such as diabetes and cardiovascular disease [10,11,68]. The upregulation of *AKR1C3*, *UCHL1* and *NQO1* has been linked with obesity previously. Thus, *AKR1C3*, a gene that encodes a member of the aldo/keto reductase superfamily, apparently induces androgen inactivation, increasing adiposity [69–71]. Furthermore, central obesity, which is more pernicious than peripheral obesity, is associated with overexpression of this gene [71]. Additionally, Svensson *et al*. [72] have shown that diet induced weight loss reduced *AKR1C3* mRNA levels in human obese subjects, and that larger adipocytes presented higher levels of this gene in comparison to smaller adipocytes. Moreover, a deubiquitinating enzyme, Ubiquitin carboxy-terminal hydrolase L1 (UCH-L1), seemed to be deficient in humans with type 2 diabetes [73], although it has been found overexpressed under hypoxic conditions and in visceral fat from humans modulating Peroxisome proliferator-activated receptor gamma (PPARγ) signaling pathway [74]. In addition, Palming *et al*. [75] have shown that *NQO1* expression, a member of the reduced Nicotinamide Adenine Dinucleotide Phosphate (NAD(P)H) dehydrogenase (quinone) family, is increased in human adipose tissue, reduced by weight loss, and correlates with adiposity, insulin sensitivity, and markers of liver dysfunction. Furthermore, the use of capsaicin as an antiobesity compound reduced the expression of *NQO1* in rats [76].

Interestingly, HSPB7 a heat shock protein, to our knowledge, has not been linked to obesity, but there are several studies reporting its association to cardiovascular disease, although its overexpression seems to be protective [77], common variants in this gene have been associated with advanced heart failure [78].

### 2.3. Under-Represented GO Biological Process Categories

#### 2.3.1. Genes Involved in Apoptosis

The apoptosis induction pathway was downregulated (*p* < 0.05), five transcripts encoding proteins involved in this inhibited pathway are: *RAD21; S100B; RHOB; PLAGL1; CIDEA*.

#### 2.3.2. Genes in Cell Control and Cell Cycle

A general downregulation of genes concerning categories of the regulation of cell cycle control/cell proliferation, and cell growth and maintenance that might reflect the change in SCAAT from obese subjects was found (Table S2).

#### 2.3.3. Genes in Signal Transcription

The transcription regulation category included fifteen genes that were downregulated (*p* < 0.05). Interestingly, and contrary to expectations, several genes involved in the JNK (c-Jun N-terminal kinases) signal transduction pathway: *FOS*, *FOSB*, and *JUN* (forming the transcription factor complex AP-1, activator protein 1) were downregulated. However, the data of the RT-PCR analysis did not confirm the *FOS* downregulation in SCAAT from obese compared to lean. In addition to this, Chazenbalk *et al.* [79] and Jones *et al.* [80] have recently reported an underexpression of *FOS* and *JUN* in women with Polycystic Ovary Syndrome, a disease often accompanied by obesity [81,82], agreeing with our results found in young male subjects. Moreover, *CITED2* a member of the cited family of nuclear regulators and *NR3C1* were downregulated (Table 2).

Additional data are given in Tables S1 and S2, which show other interesting upregulated and downregulated genes. For instance, a gene importantly upregulated is *SVEP1*, which has not been previously identified in adipose tissue. SVEP1 is a novel cell adhesion molecule that has been shown to be expressed throughout the early phases of myogenesis [83] and in cultured osteogenic cells [84]. There is no information about the function of SVEP1 in adipose tissue, but it would be interesting to address if it has a potential role in adipogenesis in obesity.

The current study, as others found in the scientific literature [85], has used RNA samples derived from adipose tissue for gene expression profile analysis focusing on obesity. This kind of study design is distinct from those in which RNA extracts prepared from separated adipocytes and stroma vascular cells (SVC) were utilized. However, it is worth noting that a study with Pima Indians, observed a similar upregulation of inflammation-related genes in both preadipocytes/stromal vascular cells and in adipocytes of adipose tissue from obese Pima Indians, demonstrating that both preadipocytes/SVC and adipocytes may play complementary roles in obesity-related inflammation [86,87]. The above-mentioned studies reported a *NR3C1* downregulation as we found in our study.

### 2.4. Association of Gene Expression with Metabolic Syndrome

Interestingly, 11 genes differentially expressed in the results of this array, such as: *ALCAM; CTSB*, *C1S*, *CITED2*, *YKL40*, *EGFL6*, *MIF*, *NR3C1*, *PALLD*, *SAA2* and *TNMD*, were found to be correlated with some relevant metabolic syndrome features considering all the enrolled subjects as a whole (Table 3).

For example, gene expression of several transcripts encoding components of the innate immune system as *C1S* and *SAA2* a proinflammatory and lipolytic adipokine, were positively associated with waist circumference (*r* = 0.69; *p* < 0.01, *r* = 0.71; *p* < 0.05, respectively). Furthermore, *C1S* correlated negatively with QUICKI (*r* = −0.51; *p* < 0.05), and HDL-cholesterol (*r* = −0.67; *p* < 0.01); and positively with triglycerides (*r* = 0.71; *p* < 0.01) and total cholesterol (*r* = 0.57; *p* < 0.05). Moreover, *SAA2* was associated positively with BMI (*r* = 0.53; *p* < 0.05).

In addition, the association of several genes related to inflammation: *ALCAM*, an adhesion molecule of the Ig superfamily, which correlates with BMI (*r* = 0.73; *p* < 0.01) waist circumference (*r* = 0.71; *p* < 0.01), QUICKI (*r* = −0.59, *p* < 0.05), total cholesterol (*r* = 0.65; *p* < 0.05), HDL cholesterol (*r* = −0.68; *p* < 0.01), and systolic BP (*r* = 0.59; *p* < 0.05) was evidenced. *CTSB* a member of the cathepsin family correlated with BMI (*r* = 0.61; *p* < 0.05), waist circumference (*r* = 0.75: *p* < 0.01). These findings involving *C1S*, *SAA2*, *ALCAM* and *CTSB* are novel to the author’s knowledge.

*YKL-40* (also known as *CHI3L1*), recently defined as a biomarker of inflammation elevated in patients with type 2 diabetes and related to insulin resistance, correlates with BMI (*r* = 0.53; *p* < 0.05), as seen in previous studies [88–90] and in contrast with Nielsen *et al.* [42]. Furthermore, *tenomodulin*, a member of the cytoskeleton involved in the fibrosis process correlated with BMI (*r* = 0.51; *p* < 0.05), as found by the group of Carlsson [63]. In this context, it has been observed that an upregulation of certain genes in the inflammatory response and cell adhesion molecules promote the recruitment of monocytes and other cells, triggering the cardiovascular disease [91].

## 3. Experimental Section

### 3.1. Experimental Subjects

Nine lean (22–33 years old) and nine obese (21–35 years old) male high fat consumers with similar physical activity patterns and matched by age were recruited, using a validated questionnaire based on self-reported questions about lifestyle and food frequency consumption as previously described [18]. In order to confirm that the amount and composition of the energy intake was >40% from fat, each subject completed a 3 day weighed food record for two weekdays and one weekend day. The food records were analyzed with a computerized program (Medisystem, SanoCare, Madrid, Spain) by a trained nutritionist. Physical activity/sedentary lifestyles were assessed (Table 1) through the number of hours per week spent sitting down, (watching TV or videos, computer games, reading or listening to music, *etc.*) on a typical work day and on a typical weekend day [92].

### 3.2. Anthropometrical Measurements and Adipose Tissue Biopsy

On the experimental day, volunteers arrived at the Clínica Universidad de Navarra after 12 h of overnight fast. Anthropometrical measurements were made by standard procedures as previously described [18]. Then, biopsies of subcutaneous abdominal periumbilical area adipose tissue (1–2 g) were performed by liposuction under local anaesthesia following an overnight fast. The samples were washed, soaked in RNA-later (Qiagen, Valencia, CA, USA) to avoid RNA degradation and then stored at −80 °C until their utilization. The protocol was approved by the Ethical Committee of the University of Navarra meeting the standards of the Declaration of Helsinki (Add 1997), and all subjects gave their written informed consent before participating in the study.

### 3.3. Blood Pressure and Measurements

Blood pressure (systolic and diastolic) was measured with a standard mercury sphygmomanometer (Minimus II, Riester, Germany) as described elsewhere [93]. Fasting blood measurements were made by standard procedures as previously described [18]. The quantitative insulin sensitivity check index (QUICKI) was determined using the inverse of the sum of the logarithms of the fasting insulin (μU/mL) and fasting glucose (mg/dL).

### 3.4. Microarray Analysis

Total RNA was isolated from each human subcutaneous fat sample as previously described [45]. Then, RNA was pooled to minimize the biological variation between the individual lean and obese subjects. Thus, 15 μg of total RNA from two pools of three lean subjects (L1 and L2) and two other from three obese individuals (O1 and O2) were used in the standard protocol from Affymetrix to label targets. These targets (biotinylated complementary RNA were hybridized to the Human HG-U133 A GeneChip arrays (Affymetrix, Santa Clara, CA, USA) at Progenika Biopharma Inc (Bilbao, Spain), using tools obtained from Affymetrix and according to the manufacturer’s protocol (Affymetrix, Santa Clara, CA, USA). Thus, a total of four array hybridizations were performed.

Differences in expression of individual genes between obese-susceptible and lean-resistant, were analyzed using Microarray Analysis Suite (MAS) 5.0 (Affymetrix, Santa Clara, CA, USA). The alteration ratios of the gene expression were represented as means of Signal Log Ratio (SLR) of the four quotients. Quotients were calculated from the gene expression for the obese subjects divided by that of the lean subjects. The “change call” criteria of Affymetrix software for several known genes related to obesity matched a call change value of 75% (“increase”: *leptin*; “decrease”: *adiponectin*). These changes were verified by RT-PCR [45]. For this reason, as a cutoff value, concordance in the different call change of 75% or more was chosen in the indications of “increase” and “decrease” for obesity-dependent changes, as previously described [94]. Then, the results from MAS were classified according to GO biological process criteria [95] and analysis of biological pathways was performed by the WebGestalt system [96], which uses the hypergeometric test to identify those pathways in which the number of identified genes exceeded the number expected (*p* < 0.05). Up and downregulated genes were analyzed separately.

### 3.5. Real-Time PCR Analysis

Differential gene expression was further confirmed by RT-PCR of a subset of genes from individual SCAAT sample cDNA (*n* = 9 in each group). Reagents for RT-PCR analysis of: *Activated leukocyte cell adhesion molecule (ALCAM)*, *Cathepsin B* (*CTSB*), *Complement component 1*, *s subcomponent* (*C1S*), *CBP/p300-interacting transactivator with ED-rich tail 2* (*CITED2*), *YKL-40* (*chitinase 3-like 1 (CHI3L1)*), *EGF-like-domain*, *multiple 6* (*EGFL6*), *v-fos FBJ murine osteosarcoma viral oncogene homolog FOS*, *Tenomodullin (TNMD)*, *macrophage migration inhibitory factor (MIF)*, *Palladin (PALLD)*, *serum amyloid A2 (SAA2)*, *nuclear receptor subfamily 3*, *group C*, *member 1 (NR3C1)* and *18S (*Assays-on-Demand, TaqMan Reverse Transcriptase reagents, and TaqMan Universal PCR Master mix) were purchased from Applied Biosystems (Foster City, CA, USA) and the experimental conditions were used according to the manufacturer’s protocol. Amplification and detection of specific products were performed with the ABI PRISM 7000HT system (Applied Biosystems). Human *18S* was used as reference to normalize the expression levels between samples allowing data to be expressed relative to *18S* rRNA, therefore compensating for any differences in reverse transcriptase efficacy, as previously described [45].

### 3.6. Statistical Analysis

Differences between the lean and obese groups were analysed by the unpaired Student’s *t* or *U* Mann Whytney’s test after testing the normality with the Kolmogorov–Smirnoff and Shapiro–Wilk tests. The Spearman correlation coefficient was used to identify related variables. Statistical analysis was performed using the SPSS 15.1 software for Windows (SPSS Inc., Chicago, IL, USA). Values of *p* < 0.05 were considered as statistically significant.

## 4. Conclusions

In summary, we characterized two groups of subjects with different susceptibility to gaining weight and developing metabolic syndrome cluster, despite both groups eating a similar amount of fat and performing the same level of physical activity. In this context, we suggested that it is not an excessive energy intake *per se* but a genetic background favoring chronic disturbance of metabolic homeostasis, which could be behind the upregulation of the proinflammatory/oxidative stress-related genes and could underlie a vulnerability to the metabolic syndrome.

## Supplementary Information



## Figures and Tables

**Table 1:** Anthropometrical and clinical parameters of volunteers.

Baseline descriptive characteristics	Lean (*n* = 9)		Obese (*n* = 9)		*p* value
	
	Mean	SE	Mean	SE	
Energy (Kcal)	2,766.7	258.7	2799.1	171.4	0.918
Fat intake (%E)	44.6	2.2	42.5	1.8	0.573
Physical activity (METs h/week)	17.5	5.1	18.0	4.4	0.945
Watching TV (METs h/week)	12.2	2.5	10.2	3.2	0.621
BMI (kg/m^2^)	23.1	0.4	34.7	1.2	0.000
Waist circumference (cm)	78.7	1.2	105.7	2.6	0.000
QUICKI	0.40	0.00	0.35	0.01	0.004
Triglycerides (mg/dL)	85.0	6.7	142.2	10.6	0.001
Total Cholesterol (mg/dL)	167.4	17.7	188.5	6.3	0.008
HDL-Cholesterol (mg/dL)	43.3	1.7	40.0	2.4	0.059
Total Cholesterol/HDL-C	3.5	0.2	5.0	0.3	0.003
Systolic BP (mmHg)	122.5	3.6	139.1	2.8	0.002
Diastolic BP (mmHg)	74.0	2.2	82.8	3.0	0.002

Abbreviations: BMI: Body mass index; QUICKI, Quantitative Insulin-Sensitivity Check Index; HDL, High-Density Lipoprotein; BP, Blood Pressure; MET, Metabolic Equivalent of Task; SE, Standard Error. Independent Student’s *t*-test or Mann-Whitney *U*-test were performed, as appropriate, depending on the results of Kolmogorov-Smirnoff and Shapiro-Wilk normality tests.

**Table 2:** Differentially expressed genes in subcutaneous abdominal adipose tissue (SCAAT) of obese *vs.* lean subjects with similar dietary and lifestyle habits.

Gene name	Gene symbol	SLR (Microarray)		Lean		Obese		RT-PCR *p* value

		Mean	SD	Mean	SD	Mean	SD	
**Upregulated**								
*Activated leukocyte cell adhesion molecule*	*ALCAM*	0.73	0.33	1.0	0.2	4.9	3.9	0.048
*Cathepsin B*	*CTSB*	0.65	0.45	1.0	0.2	1.8	0.4	0.026
*Complement component 1*, *S subcomponent*	*C1S*	0.58	0.25	1.0	0.2	2.3	0.4	0.003
*Chitinase 3-like 1 (CHI3L1) or human cartilage glycoprotein-39*	*YKL-40*	1.28	0.53	1.0	0.2	1.9	0.4	0.026
*EGF-like-domain*, *multiple 6*	*EGFL6*	3.13	0.79	1.0	0.2	2.0	0.4	0.015
*Macrophage migration inhibitory factor*	*MIF*	0.58	0.10	1.0	0.4	2.8	1.0	0.031
*Palladin*	*PALLD*	1.10	0.22	1.0	0.3	2.5	1.7	0.037
*Serum amyloid A2*	*SAA2*	0.93	0.91	1.0	1.8	6.8	4.6	0.020
*Tenomodulin*	*TNMD*	1.53	0.56	1.0	0.2	5.0	0.3	0.003
**Downregulated**								
*Cbp/p300-interacting transactivator*	*CITED2*	−1.05	0.26	1.0	0.4	0.3	0.1	0.019
*v-Fos FBJ murine osteosarcoma viral oncogene*	*FOS*	−2.75	2.10	1.0	0.2	0.9	0.4	0.998
*Nuclear receptor subfamily 3*, *group C*, *member 1*	*NR3C1*	−0.90	0.18	1.0	0.2	0.2	0.1	0.028

The alteration ratios of the gene expression are represented as means of signal log ratio (SLR) of the four quotients (see experimental Section 3.4). Quotients were calculated from the gene expression for the obese subjects divided by that of the lean subjects. Differential gene expression was further confirmed by RT-PCR of a subset of genes. SD, Standard Deviation; *n* = 9 in each group. Differences between the lean and obese groups were analysed by the unpaired Student’s *t* or *U* Mann Whytney’s test after testing the normality with the Kolmogorov-Smirnoff and Shapiro-Wilk tests.

**Table 3:** Correlation between gene expression and some relevant metabolic syndrome (MS) features.

MS features	*ALCAM*		*CTSB*		*C1S*		*CITED2*		*YKL-40*		*EGFL6*		*MIF*		*NR3C1*		*PALLD*		*SAA2*		*TNMD*	

	*r*	*p*	*r*	*p*	*r*	*p*	*r*	*p*	*r*	*p*	*r*	*p*	*r*	*p*	*r*	*p*	*r*	*p*	*r*	*p*	*r*	*p*
BMI (kg/m^2^)	0.73	**	0.61	*	0.42	–	−0.51	–	0.53	*	0.17	–	0.25	–	−0.69	*	0.46	–	0.53	*	0.51	*
Waist circumference (cm)	0.71	**	0.75	**	0.69	**	−0.52	–	0.35	–	−0.02	–	0.66	*	−0.49	–	0.56	*	0.71	*	0.34	–
QUICKI	−0.59	*	−0.34	–	−0.51	*	0.21	–	−0.15	–	−0.41	–	−0.42	–	0.73	*	−0.33	–	−0.12	–	−0.47	–
Triglycerides (mg/dL)	0.41	–	0.15	–	0.71	**	−0.27	–	0.19	–	0.56	*	0.31	–	−0.52	–	0.45	–	0.37	–	0.35	–
Total-Chol (mg/dL)	0.65	*	0.31	–	0.57	*	−0.08	–	0.25	–	0.15	–	0.36	–	−0.50	–	0.48	–	0.43	–	0.44	–
HDL-Chol (mg/dL)	−0.68	**	−0.24	–	−0.67	**	0.15	–	−0.10	–	−0.36	–	−0.22	–	0.16	–	−0.64	*	−0.28	–	−0.32	–
Systolic BP (mmHg)	0.59	*	0.39	–	0.39	–	−0.73	**	0.45	–	−0.18	–	0.12	–	−0.65	–	0.47	–	0.22	–	0.29	–
Diastolic BP (mmHg)	0.20	–	0.11	–	0.36	–	−0.78	**	0.47	–	0.25	–	−0.11	–	−0.42	–	0.37	–	0.32	–	0.09	–

Spearmann correlation was performed between gene expression (arbitrary units 2*^−ΔΔCt^*) and other parameters.

* *p* < 0.05;

** *p* < 0.01.

BMI: Body mass index; insulin sensitivity was indirectly determined by the QUICKI model. All the studied subjects were considered as a whole group, *n* = 18.
