# Supplementary material for: Differential Proinflammatory and Oxidative Stress Response and Vulnerability to Metabolic Syndrome in Habitual High-Fat Young Male Consumers Putatively Predisposed by Their Genetic Background
Source: Int J Mol Sci. 2013 Aug 22;14(9):17238–55. doi: 10.3390/ijms140917238 (PMC3794726; doi:10.3390/ijms140917238)
Supplement: Supplementary file 1 [file ijms-14-17238-s001.pdf]

Supplementary Information

Table S1. Upregulated transcripts in SCAAT of obese *vs*. lean subjects classified according to GO biological process criteria.

GO biological process	Gene symbol	Gene name	SLR		Reference sequence
			Mean	SD	
Cell cycle	*EGFL6*	EGF-like-domain, multiple 6	3.13	0.79	NM_015507
Cell growth and cell proliferation	*LTBP2*	Latent transforming growth factor beta binding protein 2	0.62	0.30	NM_000428
	PALLD	Palladin, cytoskeletal associated protein	1.10	0.22	NM_016081
	TAGLN	Transgelin	0.90	0.35	NM_003186
	ACP5	Acid phosphatase 5, tartrate resistant	1.58	0.53	NM_001611
	GPNMB	Glycoprotein (transmembrane) nmb	0.53	0.17	NM_002510
Immune and inflammation response	*CHI3L1/YKL40*	Chitinase 3-like 1 or human cartilage glycoprotein-39	1.28	0.53	NM_001276
	SPP1	Secreted phosphoprotein 1	1.78	0.43	NM_000582
	CTGF	Connective tissue growth factor	1.25	0.19	NM_001901
	TPSAB1	Tryptase alpha/beta 1	0.95	0.06	NM_003294
Chemokine receptor activity	*DARC*	Duffy blood group, chemokine receptor	0.80	0.16	NM_002036
	ALCAM	Activated leukocyte cell adhesion molecule	0.73	0.33	NM_001627
	C1S	Complement component 1, s subcomponent	0.58	0.25	NM_001734
	MIF	Macrophage migration inhibitory factor	0.58	0.10	NM_002415
	HLA-DQA1	Major histocompatibility complex, class II, DQ alpha 1	0.90	0.79	NM_002122
	DEFA1	Defensin, alpha 1, myeloid-related sequence	1.25	0.71	NM_004084
	SAA2	Serum amyloid A2	0.93	0.91	NM_030754
	HLA-DRB4	Major histocompatibility complex, class II, DR beta 4	4.05	0.51	NM_021983
	SPON2	Spondin 2, extracellular matrix protein	0.75	0.19	NM_012445
	TUBB2	Tubulin, beta 2	0.75	0.17	NM_001069
	ITGB5	Integrin, beta 5	0.68	0.37	NM_002213
	TNMD	Tenomodulin	1.53	0.56	NM_022144
	IGLC1	immunoglobulin lambda constant 1	0.68	0.66	X57812
	CD59	CD59 molecule, complement regulatory protein	0.40	0.24	NM_000611
Proteolysis and peptidolysis	*CTSB*	Cathepsin B	0.65	0.45	NM_001908
	MMP9	Matrix metallopeptidase 9	1.30	0.67	NM_004994

Table S1. *Cont.*

GO biological process	Gene symbol	Gene name	SLR		Reference sequence
			Mean	SD	
Signal transduction	*EIF5A*	Eukaryotic translation initiation factor 5A	1.70	0.98	NM_001970
	WISP2	WNT1 inducible signaling pathway protein 2	1.55	0.37	NM_003881
	CNKSR2	Connector enhancer of kinase suppressor of Ras 2	1.38	0.38	NM_014927
	CRYAB	Crystallin, alpha B	0.78	0.74	NM_001885
	MEN1	Multiple endocrine neoplasia I	0.53	0.86	NM_000244
Stress response	*NQO1*	NAD(P)H dehydrogenase, quinone 1	1.30	0.41	NM_000903
	UCHL1	Ubiquitin carboxyl-terminal esterase L1	1.10	0.58	NM_004181
	HSPB7	Heat shock 27 kda protein family, member 7	0.78	0.46	NM_014424
Transport	*SLC5A3*	Solute carrier family 5, member 3	0.53	0.38	NM_006933
	MAP1B	Microtubule-associated protein 1B	0.78	0.43	NM_005909
	SVEP1	Sushi, von Willebrand factor type A, EGF and pentraxin domain containing 1	3	1.87	NM_153366.3

SLR: Signal Log Ratio; SD: Standard Deviation.

Table S2. Downregulated transcripts in SCAAT of obese *vs*. lean subjects classified according to GO biological process criteria.

GO biological process	Gene symbol	Gene name	SLR		Reference sequence
			Mean	SD	
Cell cycle	*FOSB*	FBJ murine osteosarcoma viral oncogene homolog B	−3.38	0.26	NM_006732
	CDKN2C	Cyclin-dependent kinase inhibitor 2C (p18, inhibits CDK4)	−1.20	0.47	NM_001262
	CIDEA	Cell death-inducing DFFA-like effector a	−1.13	0.38	NM_001279
	DUSP6	Dual specificity phosphatase 6	−1.00	0.77	NM_001946
	ARMCX2	Armadillo repeat containing, X linked 2	−0.93	0.31	NM_014782
	PLAGL1	Pleiomorphic adenoma gene-like 1	−0.65	0.17	NM_002656
	CCNG2	Cyclin G2	−0.65	0.26	NM_004354
	RAD21	RAD21 homolog (S. Pombe)	−0.53	0.17	NM_006265
Cell growth and cell proliferation	*CYR61*	Cysteine-rich, angiogenic inducer, 61	−1.68	0.38	NM_001554
	S100B	S100 calcium binding protein, Beta	−0.90	0.50	NM_006272
	LRIG1	Leucine-rich repeats and immunoglobulin-like domains 1	−0.88	0.22	NM_015541
Immune and inflammation response	*CA3*	Carbonic anhydrase III, muscle specific	−2.05	0.77	NM_005181
	ZFP36	Zinc finger protein 36, C3H type, homolog (mouse)	−0.93	0.75	NM_003407
	MUT	Methylmalonyl Coenzyme A mutase	−0.80	0.73	NM_000255
	CAPN7	Calpain 7	−0.70	0.24	NM_014296
	IER2	Immediate early response 2	−0.63	0.15	NM_004907
	NID1	Nidogen 1	−0.60	0.55	NM_002508
	CXCL14	Chemokine (C–X–C motif) ligand 14	−0.60	0.35	NM_004887
	CD302	CD302 molecule	−0.53	0.13	NM_014880
	DDIT4	DNA-damage inducible transcript 4	−0.53	0.26	NM_019058
	NID2	Nidogen 2 (osteonidogen)	−0.53	0.49	NM_007361
	RGCC	Regulator of cell cycle	−0.50	0.49	NM_014059
	TCRA/TCRD	T cell receptor alpha delta locus	−0.50	0.39	NG_001332.2
	PTENP1	Phosphatase and tensin homolog pseudogene 1	−0.45	0.34	NG_001337.2
	SAT1	Spermidine/spermine N1-acetyltransferase, transcript variant 1	−0.55	0.10	NM_002970

Table S2. *Cont.*

GO biological process	Gene symbol	Gene name	SLR		Reference sequence
			Mean	SD	
Signal transduction	*FOS*	v-FOS FBJ murine osteosarcoma viral oncogene	−2.75	2.10	NM_005252
	EGR1	Early growth response 1	−1.58	0.28	NM_001964
	DDX3X	DEAD (Asp-Glu-Ala-Asp) box polypeptide 3, X-linked	−0.33	0.17	NM_001356
	CLMN	Calmin (calponin-like, transmembrane	−1.18	0.44	NM_024734
	CITED2	Cbp/p300-interacting transactivator	−1.05	0.26	NM_006079
	HLF	Hepatic leukemia factor	−0.98	0.49	NM_002126
	GNAI1	Guanine nucleotide binding protein (G protein)	−0.95	0.31	NM_002069
	NR3C1	Nuclear receptor subfamily 3, group C, member 1	−0.90	0.18	NM_000176
	SVIL	Supervillin	−0.90	0.45	NM_003174
	TJP2	Tight junction protein 2 (zona occludens 2)	−0.88	0.28	NM_004817
	TWIST1	Twist homolog	−0.88	0.28	NM_000474
	KLF4	Kruppel-like factor 4 (gut)	−0.83	0.31	NM_004235
	NRIP1	Nuclear receptor interacting protein 1	−0.80	0.44	NM_003489
	ATF2	Activating transcription factor 2	−0.75	0.26	NM_001880
	SPRY1	Sprouty homolog 1, antagonist of FGF signaling (Drosophila)	−0.70	0.29	NM_005841
	RHOB	Ras homolog gene family, member B	−0.68	0.19	NM_004040
	ANK2	Ankyrin 2	−0.68	0.22	NM_001148
	CXCR7	Chemokine (C–X–C motif) receptor 7	−0.68	0.57	NM_020311
	JUN	Jun proto-oncogene	−0.68	0.31	NM_002228
	GLUL	Glutamate-ammonia ligase (glutamine synthetase)	−0.68	0.05	NM_002065
	EIF3K	Eukaryotic translation initiation factor 3, subunit K	−0.48	0.10	NM_013234
	EIF3L	Eukaryotic translation initiation factor 3, subunit L	−0.48	0.10	NM_016091
	NR1D1	Nuclear receptor subfamily 1, group D, member 1	−0.48	0.38	NM_021724
	TIMP4	Metallopeptidase inhibitor 4	−0.48	0.45	NM_003256
	RAPGEF2	Rap guanine nucleotide exchange factor (GEF) 2	−0.38	0.10	NM_014247
	MYOD1	Myogenic differentiation 1	−0.30	0.16	NM_002478
	AGTR1	Angiotensin II receptor, type 1	−0.10	0.39	NM_000685

Table S2. *Cont.*

GO biological process	Gene symbol	Gene name	SLR		Reference sequence
			Mean	SD	
Stress response	*GHR*	Growth hormone receptor	−0.73	0.33	NM_000163
	VEGFA	Vascular endothelial growth factor A	−1.53	0.58	NM_003376
	NDRG4	NDRG family member 4	−1.50	0.64	NM_020465
	NET1	Neuroepithelial cell transforming gene 1	−0.88	0.22	NM_005863
	IGF1	Insulin-like growth factor 1 (somatomedin C)	−0.98	0.13	NM_000618
	DPYD	Dihydropyrimidine dehydrogenase	−0.35	0.29	NM_000110
	DUSP1	Dual specificity phosphatase 1	−0.85	0.79	NM_004417
	TCF7L2	Transcription factor 7-like 2 (T-cell specific, HMG-box)	−0.30	0.24	NM_030756
	HMGB2	High-mobility group box 2	−0.28	0.17	NM_002129
Transport	*SLC39A14*	carrier family 39 (zinc transporter), member 14	−0.35	0.29	NM_001128431
	SLC19A2	Solute carrier family 19 (thiamine transporter), member 2	−0.93	0.13	NM_006996

SLR: Signal Log Ratio; SD: Standard Deviation.

© 2013 by the authors; licensee MDPI, Basel, Switzerland. This article is an open access article distributed under the terms and conditions of the Creative Commons Attribution license (http://creativecommons.org/licenses/by/3.0/).
